# 30. Implementation of the PEN-FAST Penicillin Allergy Screening Tool in the Emergency Department During Medication Reconciliation

**DOI:** 10.1093/ofid/ofab466.232

**Published:** 2021-12-04

**Authors:** Satwinder Sony Kaur, David T Adams, Brittany Parker

**Affiliations:** 1 Memorial Hermann-Texas Medical Center , Houston, Texas; 2 Texas Health Harris Methodist Hospital Fort Worth, Fort Worth, Texas

## Abstract

**Background:**

The purpose of this study is to implement the PEN-FAST Penicillin Allergy Screening Tool in the emergency department to identify low risk patients with inappropriate penicillin-related allergies to transition them to a beta-lactam. Newly published, validated, penicillin allergy clinician decision tool (PEN-FAST) allows healthcare providers to identify low risk penicillin allergies with a negative predictive value of 96%. This quick, five question clinical decision tool allows healthcare providers and antimicrobial stewardship programs to identify patients who would also test negative if a formal penicillin allergy test was performed, making the process to confidently identify inappropriately labeled penicillin-related allergies more efficient.

**Methods:**

During routine medication reconciliations, pharmacists will identify patients who have a documented penicillin-related allergy in the EMR and use the PEN-FAST screening tool. Patients meeting inclusion criteria will have their penicillin-related allergy updated in the EMR based upon their assessed risk of very low, low, moderate, or high. The primary outcomes for this study are the percentage of patients screened that were classified as “very low and low risk” and percentage penicillin-related allergies updated. The secondary outcomes are the percentage of patients that required antibiotic therapy (post-allergy update) that were transitioned to a beta-lactam, inpatient broad-spectrum antibiotic usage before and after allergy update, and time spent interviewing each patient.

**Results:**

A total of 59 patients were interviewed using the PEN-FAST Tool. The results for the primary outcomes indicate 92% (n=54) of patient allergies updated in the EMR, 24% (n=13) of patients classified as “very low risk” and 34% (n=18) of patients classified as “low risk”. Results for the secondary outcome showed out of the 36 patients that were on non-beta lactams during allergy update, 72% (n=26) of those patients were transitioned to a beta-lactam. The average time to complete the PEN-FAST Tool was 4.2 minutes.

**Conclusion:**

The results of this study support the use of the PEN-FAST Tool in efficiently updating patient’s allergies in the EMR and identifying low risk patients who may be eligible for beta-lactam therapy.

**Disclosures:**

**All Authors**: No reported disclosures

